# Prediction models and associated factors on the fertility behaviors of the floating population in China

**DOI:** 10.3389/fpubh.2022.977103

**Published:** 2022-09-09

**Authors:** Xiaoxia Zhu, Zhixin Zhu, Lanfang Gu, Liang Chen, Yancen Zhan, Xiuyang Li, Cheng Huang, Jiangang Xu, Jie Li

**Affiliations:** ^1^Department of Epidemiology & Biostatistics, and Center for Clinical Big Data and Statistics, Second Affiliated Hospital, College of Medicine, Zhejiang University, Hangzhou, China; ^2^Zhejiang University Library, Zhejiang University, Hangzhou, China

**Keywords:** floating population, fertility behaviors, prediction, artificial neural network, logistic regression, associated factors

## Abstract

The floating population has been growing rapidly in China, and their fertility behaviors do affect urban management and development. Based on the data set of the China Migrants Dynamic Survey in 2016, the logistic regression model and multiple linear regression model were used to explore the related factors of fertility behaviors among the floating populace. The artificial neural network model, the naive Bayes model, and the logistic regression model were used for prediction. The findings showed that age, gender, ethnic, household registration, education level, occupation, duration of residence, scope of migration, housing, economic conditions, and health services all affected the reproductive behavior of the floating population. Among them, the improvement duration of post-migration residence and family economic conditions positively impacted their fertility behavior. Non-agricultural new industry workers with college degrees or above living in first-tier cities were less likely to have children and more likely to delay childbearing. Among the prediction models, both the artificial neural network model and logistic regression model had better prediction effects. Improving the employment and income of new industry workers, and introducing preferential housing policies might improve their probability of bearing children. The artificial neural network and logistic regression model could predict individual fertility behavior and provide a scientific basis for the urban population management.

## Introduction

The reform and opening-up policy implemented in 1979 promoted China's economic development, and the shifting population driven by the booming economy also expanded swiftly. The floating population referred to new industry workers without local household registration. It was a concept under the household registration system in China (1). According to a report on the development of the floating population in China, the total number reached 121 million in 2000, accounting for 10 percent of the whole country population at the time, and increased to 245 million in 2016 (2). Most new industry workers moved from rural areas to urban areas or from central and western regions to eastern coastal areas for better job opportunities and living conditions (3).

According to the 2010 census in China, 53.6 percent of the floating population was born in 1980 or later, indicating a high proportion of new industry workers in their reproductive age (15–49 years) (4). The urban fertility rate has been below the replacement level since 1990 in China (5). However, the fertility rate of the floating population was lower than that of those living in both rural and urban areas (4). In the context of low fertility in China, decreasing birth rates would lead to labor constraints (6), economic slowdown (7), lack of innovation, and population aging (8). The floating population was an important labor force in urbanization construction (9). Promoting their fertility behaviors can well-alleviate their poor psychological and social health (10), poor sense of belonging (11, 12), and poor understanding of reproductive health (13), which are closely related to the stability and development of cities. An analysis of married women in China between 1980 and 1992 showed that residence, education level, and coincident marriage affected the first birth interval (14). A study on willingness of the floating population to have a second child in Hunan Province found that the relevant factors of fertility willingness included gender, age, occupation, education level, and marital status (15). Logistic regression, neural networks, and other machine learning models had been used to predict the birth results of pregnant women (16) and live birth results of embryos (17). However, there was still a lack of model research used to predict the fertility behavior of the floating population.

The one-child policy was enacted in 1979 to slow population growth at a time when productivity in China was relatively low, and its population was growing too fast (18). Violators of the policy, which was mainly enforced in cities and densely populated rural areas, could be fined and forced to undergo abortions or sterilizations (19). For nearly 40 years, late marriage, late childbirth, and strict population control became the main tone of fertility policy in the long term. However, with the economic development, the fertility level of China continued to be low, resulting in the imbalance of gender ratio, the weakening of the demographic dividend (20), and the acceleration of population aging (21), which made the transition of fertility policy urgent. On 29 October 2015, China implemented the universal two-child policy (20). However, the response of young couples to the “two-child policy” was not positive, and their willingness to have a second child was not high (22). By 2018, the birth rate of China dropped to its lowest level in 7 decades (23). China might be entering an era of negative population growth, with serious demographic and economic consequences (24). So China introduced the three-child policy in 2021 (25). Population trends were usually defined by fertility rates, which continued to increase after reaching replacement fertility rates (26). In the context of the low fertility rate of China, encouraging marriage and childbearing could increase the fertility rate. The proportion of newborns would gradually increase, while the proportion of the elderly would correspondingly decrease, alleviating the degree of population aging (27). The increase in the fertility rate could provide support for future labor stock, and the goal of sustainable economic development would be achieved (28, 29). The demographic dividend brought by the large proportion of the working-age population could be extended (30, 31).

The Chinese government has been encouraging couples to have more children to curb negative population growth and the aging population, but the implementation of the “two-child policy and three-child policy” requires the cooperation of families and individuals. Given the floating population, a group with a low fertility rate, this study explored the factors affecting the fertility behavior of the floating population, which could be helpful for relevant departments to formulate corresponding policies and measures to promote their fertility behavior, increase the future labor population of the city, and accelerate its construction and development.

According to the 14th Five-Year Plan of the Communist Party of China Central Committee, it is necessary to strengthen the construction of the digital society and digital government and improve the digital intelligence of public services and social governance. Urban population management includes the management of the floating population, family planning, and the quantity and quality of citizens. It could further strengthen the digital construction by relying on the original population information management system. By comparing the effects of three kinds of mathematical models applied to the individualized prediction of the fertility behavior of the floating population, this study selected scientific models to help relevant departments predict the potential population increment brought by new industry workers after they settled down in the local area, identify the individuals with low fertility possibility of the floating population, and take corresponding measures.

## Data and methods

### Data source and sample

The data used in this study were obtained from the China Migrants Dynamic Survey in 2016, which used a stratified three-stage random sample proportional to the population and collected information in the form of anonymous questionnaires (32). It was a large-scale national sample survey of the floating population conducted by the National Health Commission of China, covering 31 provinces (autonomous regions and municipalities directly under the central government) and the Xinjiang Production and Construction Corps, where the floating population was highly concentrated, with a sample size of nearly 200,000 households per year. The data covered basic information about the floating population and family members, the extent and length of migration, employment and social security, income and expenditure, residence, basic public health services, marriage, and family planning services and management. This data set was the secondary data collected from the questionnaire survey of the floating population. After removing the samples with unreasonable data and blank data, 168,993 valid questionnaires were obtained. The analyses were in an anonymized form and, consequently, would not be offensive to any individual or community.

### Dependent variable

In the research on the correlative factors of fertility behavior, since the data did not meet the conditions of the ordered multi-category logistic regression, it was divided into two binary logistic regressions. In logistic regression of all samples, the question “Do you have children?” was the outcome variable. Then, the samples with children were screened out, and a logistic regression analysis was conducted with the question “Do you have two or more children?” as the outcome variable. The dependent variable was a categorical variable, where “yes” was marked as “1” and “no” as “0.”

A multiple linear regression model was applied to study the related factors of the age of first childbearing and birth spacing of the floating population. The time of first birth and the birth interval were used as the dependent variables, which were the continuous variables.

### Independent variable

The basic information of the respondents, such as gender and age, were generally included in the model as control variables (33). Some studies suggested that the education level (34) and occupation (35) might affect the fertility behavior of residents. The object of observation in this study was the floating population, so the scope of migration and the duration of residence were also worth noting. Studies showed that new industry workers could change the original fertility pattern and move closer to the fertility behavior of residents in the destination (36, 37). The precondition for new industry workers to settle down was to acquire sufficient material basis, which was closely related to new industry workers' occupation, income (35), and housing (38). In addition, some economists believed that the introduction of social insurance might reduce the population's fertility rate (39). The involvement of healthcare services was required during the reproductive process (40).

Therefore, the independent variables in this study were divided into four aspects: personal information, migration situation, economic conditions, and social services. The study encoded the relevant variables (Table 1). Personal information included gender, ethnic group, registered permanent residence, education level, and occupation. The migration situation included the duration of residence after migration and the migration range of the investigation object. Economic conditions were measured by the level of the city the respondents lived in, their monthly income in the past year, and their real estate, which was measured by whether they bought a house locally. Social services referred to those obtained by the subjects themselves, including insurance services and health services. The former included whether to participate in endowment insurance, unemployment insurance, industrial injury insurance, maternity insurance, and medical insurance. The latter referred to the establishment of residents' health records and whether they have received health education related to occupational diseases, infectious diseases, and mental diseases.

**Table 1:** Coding of categorical variables.

**Variable**	**Code**
Duration of settlement	<1 year = 1, 1–2 years = 2, 3–4 years = 3, 5–9 years = 4, 10–14 years = 5, 15–19 years = 6, 20–29 years = 7, ≥30 years = 8^*^
Scope of migration	Across the county = 1, Across the city = 2, Across the province/nation = 3^*^
City	Non-first-tier cities = 0, First-tier cities = 1^*^
Gender	Male = 0, Female = 1^*^
Ethnic	Non-Han = 0, Han = 1^*^
Household registration	Non-agriculture = 0, Agriculture = 1^*^
Marital status	Unmarried = 0, Married = 1^*^
Education level	No formal education = 1, Primary school = 2, Junior high school = 3, High school = 4, Junior college = 5, Undergraduate = 6, Postgraduate = 7^*^
Occupation status	Unemployed = 1, Employee = 2, Employer = 3, Self-employed worker = 4, Blue-collar worker = 5^*^
Housing	Rent or others = 0, Self-occupation = 1^*^
Having children	No = 0, Yes = 1
Having two or more children	No = 0, Yes = 1

* Control group.

### Methods

This study grasped the overall distribution characteristic of the floating population based on the related statistical descriptions. For univariate analysis, logistic regression and multiple linear regression models were used to analyze the influencing factors of fertility behaviors.

In the univariate analysis, the sample was grouped according to whether or not they had children. For the continuous variables with a non-normal distribution and the ordered categorical variable, the rank-sum test was used for comparison between groups. If the independent variable was an unordered categorical variable, the chi-square test was used for comparison between groups.

The aforementioned statistically significant associated factors were incorporated into the logistic regression model for multivariate analysis of fertility behavior. Logistic regression was often used to analyze the related factors of dichotomous outcomes (41, 42):


(1) $$\begin{array}{l} \log\left(\frac{\mathrm{Pr}\left(y=1\right)}{1-\mathrm{Pr}\left(y=1\right)}\right)=\alpha_{0}+b_{1}x_{1}+b_{2}x_{2}+\cdots +b_{n}x_{n} \end{array}$$

where *y* = 1 means “yes” and *y* = 0 means “no.” *x*_1_, *x*_2_, ⋯, *x*_*n*_ represent the n independent variables in this study; *b*_1_, *b*_2_, ⋯, *b*_*n*_ are the coefficients of each variable; and *e*^*b*^ is equal to the odds ratio (OR). The estimated effect was expressed by OR with 95% confidence interval (CI).

When studying the associated factors of the age of the first birth and birth interval, the multiple linear regression model was established (43–45) with the associated factors as independent variables, and the age of the first birth or birth interval as dependent variables.

According to the aforementioned factors, the artificial neural network and naive Bayes models could be established. The first M = 90,000 samples were selected as the training set, and the remaining samples as the test set. The correlation coefficients of each model were trained to the optimal by using the training set.

**Figure 1:**
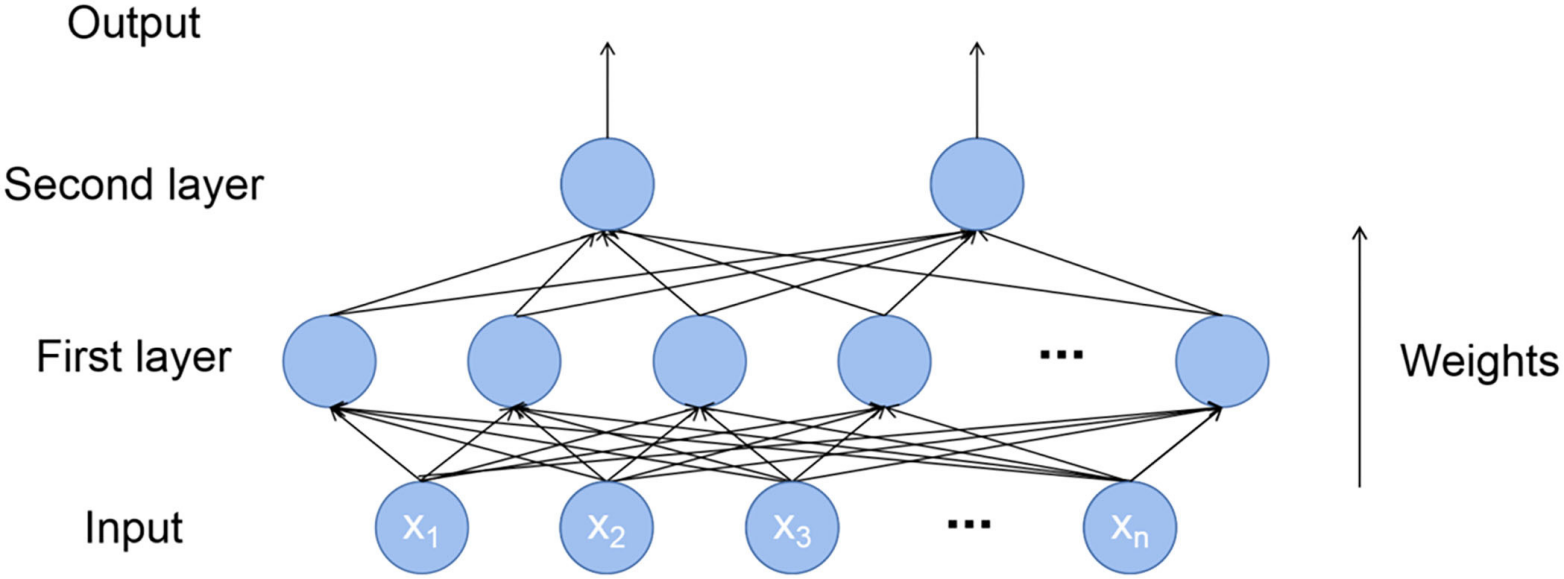
Structure of artificial neural network model (ANN).

The artificial neural network (ANN) (46) could be regarded as the simulation of the human brain nervous system. Dendrites were responsible for receiving input signals, and neurons were responsible for processing input signals. Then, they were transmitted to the next layer of neurons through synapses and continued to output after processing. The ANN model constructed in this study included input, two-layer activation function (hyperbolic tangent S-shaped function and linear function), and output (Figure 1).

The naive Bayes model (47, 48) was based on the Bayes theorem to calculate the possibility of each outcome in the case of fixed features to select the outcome with the highest possible as the predicted value. The logistic regression model (49) could estimate the probability that samples with various attribute values belonging to a certain category. Logistic regression used the likelihood function as the training function, and the maximum likelihood estimate obtained was the predicted value of model coefficients (50).

For each model with the best parameters obtained by training, feature vectors of the test set were inputted to output its prediction results, and the accuracy rate (ACC), precision rate (PRE), and recall rate (REC) of the model were calculated to measure the prediction effects of models to select the optimal model.

where TP is the number of true-positive cases, TN is the number of true-negative cases, FP is the number of false-positive cases, and FN is the number of false-negative cases.

### Statistical analysis methods

Continuous data with normal distribution were described by the mean and standard deviation. Continuous data with non-normal distribution were represented by the median and inter-quartile range (IQR). Classified data were described by using relative numbers. The rank-sum test of independent samples was used to process univariate analysis of continuous data with the non-normal distribution. Logistic regression and multiple linear regression models were used to analyze the related factors of fertility behaviors. The univariate analysis and multivariate analysis were processed by IBM SPSS Statistics 24. The artificial neural network and naive Bayes models could be conducted by MATLAB R2020a. The *P* ≤ 0.05 was considered statistically significant.

## Results

### Basic information

A total of 168,993 valid questionnaires were obtained in this study. The average age of the subjects was 39 years, with an inter-quartile range of 15 years. The local average monthly income in the previous year was 5500 CNY, with an inter-quartile range of 4000 CNY. The average insurance points was 2, with an inter-quartile range of 1. The average health service points was 6, with an inter-quartile range of 6. Descriptive statistics about the geographic location and demographic characteristics showed that 82.19% of subjects were residents from rural zones; 52.12% were male; 83.07% were married; 91.78% were Han; 61.71% had a junior high school education or less; 73.86% of the new industry workers had been away for <10 years; 46.95% were employees; 48.40% of the new industry workers crossed provinces or nations; and 72.32% of new industry workers rent houses in their city of residence (Table 2).

**Table 2:** Basic information of the floating population.

**Variables**		***n***	**Constituent**
			**ratio (%)**
Age			39 (15)^*^
Gender	Male	88,085	52.12
	Female	80908	47.88
Ethnic	Non-han	13,883	8.22
	Han	155,110	91.78
Household	Non-agriculture	30,106	17.81
	Agriculture	138,887	82.19
Marital status	Unmarried	28,604	16.93
	Married	140,389	83.07
Education level	No formal education	3,114	1.84
	Primary school	21,735	12.86
	Junior high school	79,443	47.01
	High school	37,680	22.30
	Junior college	16,509	9.77
	Undergraduate	9,704	5.74
	Postgraduate	808	0.48
Occupation	Unemployed	30,828	18.24
	Employee	79,349	46.95
	Employer	12,042	7.13
	Self-employed worker	44,213	26.16
	Blue collar worker	2,561	1.52
Duration of settlement (year)	<1	15,480	9.16
	1-	30,538	18.07
	3-	30,803	18.23
	5-	47,992	28.40
	10-	23,616	13.97
	15-	13,074	7.74
	20-	6,858	4.06
	30-	632	0.37
Scope of migration	Across the county	29,767	17.61
	Across the city	57,431	33.98
	Across the province/nation	81,795	48.40
Settlement	Non-first-tier cities	150,995	89.35
	First-tier cities	17,998	10.65
Housing	Rent or others	122,215	72.32
	Self-occupation	46,778	27.68
Monthly income			5,500 (4,000)^*^
Insurance services			2 (1)^*^
Health services			6 (6)^*^

* Value with an asterisk was median (IQR).

### Univariate analysis

The findings showed that the distribution of the variables mentioned earlier was different between the populace without children and with children, and the difference was statistically significant, including age (u = −215.36, *P* < 0.05), monthly income (u = −78.39, *P* < 0.05), insurance services (u = −4.91, *P* < 0.05), and health services (u = −7.03, *P* < 0.05). The duration of settlement (*u* = −88.48, *P* < 0.05), scope of migration (*u* = −13.48, *P* < 0.05), settlement (χ^2^ = 33.47, *P* < 0.05), ethnic (χ^2^ = 199.40, *P* < 0.05), household (χ^2^ = 374.25, *P* < 0.05), marital status (χ^2^ = 106641.67, *P* < 0.05), education (*u* = −133.30, *P* < 0.05), occupation (*u* = −49.16, *P* < 0.05), and housing (χ^2^ = 1718.00, *P* < 0.05) statistically correlated with fertility behaviors of the floating population (Table 3).

**Table 3:** Univariate analysis on fertility behavior of the floating population.

**Variable**		**Having children**	**Childless**	**Statistic**	**P**
Age		45 (13)	30 (8)	−215.36^*^	<0.001
Gender	Male	31,008	57,077	0.77	0.379
	Female	26,545	54,363		
Ethnic	Non-han	5,234	8,649	199.40	<0.001
	Han	52,319	102,791		
Household registration	Non-agriculture	5,897	24,209	374.25	<0.001
	Agriculture	51,656	87,231		
Marital status	Unmarried	189	28,415	106,641.67	<0.001
	Married	57,364	83,025		
Education level	No formal education	2,068	1,046	−133.30^*^	<0.001
	Primary school	12,736	8,999		
	Junior high school	31,557	47,886		
	High school	8,722	28,958		
	Junior college	1,771	14,738		
	Undergraduate	641	9,063		
	Postgraduate	58	750		
Occupation	Unemployed	11,028	19,800	−49.16^*^	<0.001
	Employee	19,873	59,476		
	Employer	4,918	7,124		
	Self-employed worker	20,877	23,336		
	Blue collar worker	857	1,704		
Duration of settlement (year)	<1	3,147	12,333	−88.48^*^	<0.001
	1-	6,946	23,592		
	3-	8,843	21,960		
	5-	17,088	30,904		
	10-	10,662	12,954		
	15-	6,699	6,375		
	20-	3,809	3,049		
	30-	359	273		
Scope of migration	Across the county	10,102	19,665	−13.48^*^	<0.001
	Across the city	17928	39503		
	Across the province/nation	29,523	52,272		
Settlement	Non-first-tier cities	52,331	98,664	33.47	<0.001
	First-tier cities	5,222	12,776		
Housing	Rent or others	42,871	79,344	1,718.00	<0.001
	Self-occupation	14,682	32,096		
Monthly income		5,800 (4,000)	4,500 (4,000)	−78.39^*^	<0.001
Insurance services		2 (1)	2 (3)	−4.91^*^	<0.001
Health services		6 (5)	6 (6)	−7.03^*^	<0.001

* Value with an asterisk was u *value*, and the others were χ^2^ value.

### Multivariate analysis

The number of biological children born in the floating population was taken as the outcome variable in this model. However, this model did not pass the test of parallel lines. Therefore, two binary logistic models were chosen to analyze the related factors. In the model of one-birth behavior, the sample range was all the respondents, and the model was established with whether they had biological children as the outcome variable. The survey scope in the model of the second-child fertility behavior was all the survey subjects who had children, and the model was established with whether they were to have a second child as the dependent variable.

Factors related to fertility behavior of the floating population include age (χ^2^ = 2578.01, *P* < 0.05), gender (χ^2^ = 62.07, *P* < 0.05), ethnic group (χ^2^ = 27.22, *P* < 0.05), household registration (χ^2^ = 156.61, *P* < 0.05), marital status (χ^2^ = 15581.80, *P* < 0.05), education level (χ^2^ = 1908.52, *P* < 0.05), occupation (χ^2^ = 308.26, *P* < 0.05), duration of residence (χ^2^ = 1355.19, *P* < 0.05), scope of migration (χ^2^ = 91.13, *P* < 0.05), settlement (χ^2^ = 107.82, *P* < 0.05), and monthly household income (χ^2^ = 109.53, *P* < 0.05) (Table 4). In particular, female new industry workers were more likely to have children than men (OR = 0.85, 95% CI: 0.81–0.88). The odds of new industry workers having children increased with age (OR = 1.08, 95% CI: 1.077–1.084). The odds of non-Han new industry workers bearing children were less than those of Han new industry workers (OR = 0.83, 95% CI: 0.77–0.89). The odds of the new industry workers with non-agriculture household registration had less active reproductive behavior than those with agriculture household registration (OR = 0.72, 95% CI: 0.68–0.76). In addition, new industry workers with better economic conditions (OR = 1.24, 95% CI: 1.19–1.29) were more likely to have children. Fertility behavior and education level show an inverted U-shaped distribution. Under junior high school, the higher the education level, the more the childbirth. However, for the new industrial workers whose education level is junior high school or above, the higher the education level, the fewer the childbirth. New industrial workers with junior high school education had 6.07 times as many children as those with postgraduate (OR = 6.07, 95% CI: 5.06–7.29).There is no statistical difference in the fertility behavior of employee and blue-collar workers (OR = 1.08, 95% CI: 0.93–1.25), but the fertility behavior of employers (OR = 1.29, 95% CI: 1.09–1.52), self-employed workers (OR = 1.77, 95% CI: 1.51–2.07), and the unemployed (OR = 1.41, 95% CI: 1.21–1.65) is higher than that of blue-collar workers. The number of children born to new industrial workers living in non-first-tier cities was 1.4 times that of those living in first-tier cities (OR = 1.40, 95% CI: 1.31–1.49) (Table 4).

**Table 4:** Associated factors of the one-birth fertility behaviors of floating population.

**Variable**		***b***	** $s_{\bar{x}}$ **	**Waldχ^2^ Value**	***P***	**OR (95%CI)**
Age		0.077	0.002	2,578.014	0.000	1.08 (1.08–1.08)^*^
Gender						
	Female					1.00
	Male	−0.169	0.021	62.065	0.000	0.85 (0.81–0.88)
Ethnic						
	Han					1.00
	Non-han	−0.190	0.036	27.222	0.000	0.83 (0.77–0.89)
Household registration						
	Agriculture					1.00
	Non-agriculture	−0.331	0.026	156.607	0.000	0.72 (0.68–0.76)
Marital status						
	Married					1.00
	Unmarried	−5.686	0.046	15,581.804	0.000	0.00 (0.00–0.00)$
Education				1,908.521	0.000	
	Postgraduate					1.00
	Undergraduate	0.302	0.090	11.162	0.001	1.35 (1.13–1.62)
	Junior college	0.644	0.091	50.402	0.000	1.91 (1.59–2.28)
	High school	1.213	0.092	174.238	0.000	3.36 (2.81–4.03)
	Junior high school	1.804	0.093	376.859	0.000	6.07 (5.06–7.29)
	Primary school	1.678	0.101	278.739	0.000	5.36 (4.40–6.52)
	Uneducated	0.925	0.127	53.000	0.000	2.52 (1.97–3.24)
Occupation				308.264	0.000	
	Blue-collar worker					1.00
	Self-employed worker	0.571	0.080	51.170	0.000	1.77 (1.51–2.07)
	Employer	0.253	0.085	8.763	0.003	1.29 (1.09–1.52)
	Employee	0.075	0.077	0.960	0.327	1.08 (0.93–1.25)
	Unemployed	0.345	0.080	18.503	0.000	1.41 (1.21–1.65)
Duration of residence (year)				1,355.192	0.000	
	≥30					1.00
	20–29	0.583	0.257	5.137	0.023	1.79 (1.08–2.97)
	15–19	0.709	0.250	8.022	0.005	2.03 (1.24–3.32)
	10–14	0.741	0.248	8.949	0.003	2.10 (1.29–3.41)
	5–9	0.395	0.247	2.562	0.109	1.48 (0.92–2.41)
	3–4	0.140	0.247	0.322	0.570	1.15 (0.71–1.87)
	1–2	−0.218	0.247	0.781	0.377	0.80 (0.50–1.30)
	<1	−0.504	0.248	4.134	0.042	0.60 (0.37–0.98)
Scope of migration				91.127	0.000	
	Across the province/nation					1.00
	Across the city	−0.011	0.024	0.227	0.634	0.99 (0.94–1.04)
	Across the county	0.269	0.031	73.556	0.000	1.31 (1.23–1.39)
Settlement						
	First-tier cities					1.00
	Non-first-tier cities	0.336	0.032	107.818	0.000	1.40 (1.31–1.49)
Housing						
	Self-occupation					1.00
	Rent or others	−0.002	0.024	0.010	0.920	1.00 (0.95–1.05)
Monthly income (*y*10^4^*yuan*)		0.217	0.021	109.530	0.000	1.24 (1.19–1.29)
Insurance services		0.001	0.006	0.056	0.813	1.00 (0.99–1.01)
Health services		0.004	0.002	3.288	0.070	1.00 (1.00–1.01)
Constant		−2.735	0.286	91.234	0.000	-

* 1.081 (1.077–1.084), $0.003 (0.003–0.004).

Further analysis findings showed that the migrant population with non-agricultural household registration has about half the number of second children as the migrant population with agricultural household registration (OR = 0.51, 95% CI: 0.49–0.53). New industry workers with lower education levels were more motivated to have a second child. Age (OR = 1.04, 95% CI: 1.040–1.043) and household income (OR = 1.07, 95% CI: 1.05–1.09) were positively correlated with the likelihood of having a second child among the floating population. Meanwhile, the odds of the non-Han floating population giving birth to a second child was 1.42 times that of the Han floating population (OR = 1.42, 95% CI: 1.36–1.49). New industry workers living in non-first-tier cities were more likely to have a second child than those dwelling in first-tier cities (OR = 1.12, 95% CI: 1.08–1.17) (Table 5).

**Table 5:** Associated factors of the two-children fertility behaviors of floating population.

**Variable**		***b***	** $s_{\bar{x}}$ **	**Waldχ^2^ Value**	***P***	**OR (95%CI)**
Age		0.041	0.001	3,420.320	0.000	1.04 (1.04–1.04)^*^
Gender						
	Female					1.00
	Male	0.021	0.013	2.620	0.106	1.02 (1.00–1.05)
Ethnic groups						
	Han					1.00
	Non-han	0.352	0.023	234.117	0.000	1.42 (1.36–1.49)
Household registration						
	Agriculture					1.00
	Non-agriculture	−0.672	0.019	1,194.058	0.000	0.51 (0.49–0.53)
Marital status						
	Married					1.00
	Unmarried	−0.365	0.095	14.826	0.000	0.69 (0.58–0.84)
Education				2,402.741	0.000	
	Postgraduate					1.00
	Undergraduate	−0.241	0.149	2.600	0.107	0.79 (0.59–1.05)
	Junior college	0.051	0.146	0.123	0.726	1.05 (0.79–1.40)
	High school	0.518	0.145	12.803	0.000	1.68 (1.26–2.23)
	Junior high school	0.925	0.145	40.764	0.000	2.52 (1.90–3.35)
	Primary school	1.277	0.146	76.648	0.000	3.59 (2.69–4.77)
	Uneducated	1.528	0.152	101.060	0.000	4.61 (3.42–6.21)
Occupation				833.195	0.000	
	Blue-collar worker					1.00
	Self-employed worker	0.242	0.051	22.037	0.000	1.27 (1.15–1.41)
	Employer	0.181	0.055	10.909	0.001	1.20 (1.08–1.33)
	Employee	−0.183	0.051	12.745	0.000	0.83 (0.75–0.92)
	Unemployed	−0.017	0.052	0.105	0.745	0.98 (0.89–1.09)
Duration of residence (year)				1,137.891	0.000	
	≥30					1.00
	20–29	0.233	0.092	6.477	0.011	1.26 (1.06–1.51)
	15–19	0.337	0.090	14.047	0.000	1.40 (1.18–1.67)
	10–14	0.234	0.089	6.861	0.009	1.26 (1.06–1.50)
	5–9	0.024	0.089	0.075	0.785	1.03 (0.86–1.22)
	3–4	−0.155	0.089	3.005	0.083	0.86 (0.72–1.02)
	1–2	−0.314	0.090	12.318	0.000	0.73 (0.61–0.87)
	<1	−0.228	0.091	6.220	0.013	0.80 (0.67–0.95)
Scope of migration				132.464	0.000	
	Across the province/nation					1.00
	Across the city	−0.161	0.014	127.673	0.000	0.85 (0.83–0.88)
	Across the county	−0.110	0.017	40.266	0.000	0.90 (0.87–0.93)
Settlement						
	First-tier cities					1.00
	Non-first-tier cities	0.117	0.022	28.577	0.000	1.12 (1.08–1.17)
Housing						
	Self-occupation					1.00
	Rent or others	0.294	0.014	422.766	0.000	1.34 (1.30–1.38)
Monthly income (×10^4^*yuan*)		0.071	0.010	50.731	0.000	1.07 (1.05–1.09)
Insurance services		−0.005	0.005	1.060	0.303	1.00 (0.99–1.00)
Health services		−0.002	0.001	2.417	0.120	1.00 (1.00–1.00)
Constant		−3.033	0.182	278.380	0.000	-

* 1.042 (1.040–1.043).

Related independent variables were included in the multiple linear regression model, and it was found that there was no statistical relationship between monthly income and outcome variables. The factors that were positively correlated with the age of the first childbearing were insurance, health service, age, education, and housing property (Table 6). The age of first birth increased by 0.98 (95% CI: 0.95–1.00) years on average for each rank of education. The duration of settlement after migration (b = −0.03, *P* < 0.05) and the migration scope (b = −0.07, *P* < 0.05) were negatively correlated with the age of the first childbearing significantly. The first childbearing age of new industry workers living in first-tier cities was 0.338 years later than that of non-first-tier cities on average. The initial childbearing age of agricultural accounts was 0.62 (95% CI: 0.56–0.67) years earlier than that of non-agricultural accounts. Han new industry workers had one child 0.35 (95% CI: 0.28–0.43) years earlier than non-Han new industry workers on average. The age of the first birth of the female floating population is 1.49 (95% CI: 1.45–1.53) years earlier than that of the male floating population.

**Table 6:** Associated factors of age at first childbearing and birth interval of floating population.

	**Age at first childbearing**					**Birth interval**				
	***b***	** $s_{\bar{x}}$ **	***t***	***P***	**95%CI**	***b***	** $s_{\bar{x}}$ **	***t***	***P***	**95%CI**
Constant	19.882	0.098	202.133	0.000	19.69–20.08	2.721	0.142	19.223	0.000	2.44–3.00
Age (year)	0.090	0.001	80.190	0.000	0.089–0.092	0.016	0.002	10.057	0.000	0.01–0.02
Gender	−1.492	0.021	−72.791	0.000	−1.53– −1.45	0.065	0.029	2.214	0.027	0.01–0.12
Ethnic groups	−0.351	0.038	−9.234	0.000	−0.43– −0.28	0.430	0.050	8.580	0.000	0.33–0.53
Household	−0.615	0.030	−20.422	0.000	−0.67– −0.56	0.128	0.050	2.555	0.011	0.03–0.23
Education	0.976	0.012	79.792	0.000	0.95–1.00	0.100	0.019	5.309	0.000	0.06–0.14
Duration of settlement	−0.034	0.007	−4.915	0.000	−0.05– −0.02	0.120	0.009	12.909	0.000	0.10–0.14
Scope of migration	−0.066	0.014	−4.746	0.000	−0.09– −0.04	−0.054	0.020	−2.766	0.006	−0.09– −0.02
Settlement	0.338	0.035	9.599	0.000	0.27–0.41	−0.186	0.052	−3.607	0.000	−0.29– −0.09
Monthly income(×10^4^ *yua*n)	−0.005	0.005	−0.906	0.365	−0.02–0.01	0.001	0.005	0.095	0.924	−0.01–0.01
Housing	0.112	0.023	4.815	0.000	0.07–0.16	0.272	0.034	7.997	0.000	0.21–0.34
Insurance services	0.097	0.007	14.442	0.000	0.08–0.11	0.044	0.011	3.995	0.000	0.02–0.07
Health services	0.009	0.002	3.834	0.000	0.00–0.01	0.011	0.003	3.301	0.001	0.00–0.02

Insurance, health service, the duration of settlement after migration, age, and education were positively correlated with birth interval. In addition, the interval between multiple births of the floating population living in first-tier cities was 0.19 (95% CI:0.09–0.29) years shorter than that living in non-first-tier cities on average. The range of migration was a significant negative correlation factor, and the birth interval decreased by 0.05 (95% CI: 0.02–0.09) years for every one unit of migration scope increase (Table 6).

### Prediction model

The statistically significant factors mentioned previously were incorporated into the prediction models of fertility behavior of the floating population. A total of 90,000 samples were retained as training data sets to fit the models, and the remaining samples were used as validation data sets to measure the prediction accuracy of the models. The results showed that the accuracy of the naive Bayes model was slightly inferior to that of the artificial neural network and logistic regression models. The artificial neural network and logistic regression models had better prediction effects, with an accuracy of 93.3% and a recall rate higher than 92.0% (Table 7). Therefore, it was more accurate to predict the fertility behavior of the floating population by using the artificial neural network model and the logistic models, which included the independent variables of personal status, the duration of settlement after migration, migration scope, economic conditions, and social services.

**Table 7:** Comparison of the prediction effect on three models.

**Model**	**ACC**	**PRE**	**REC**
Artificial neural network	0.933	0.920	0.997
Naive bayes	0.909	0.933	0.947
Logistic regression	0.933	0.921	0.996

## Discussion

As the total fertility rate of China had been declining, the family planning policy was changed into a two-child policy and, subsequently, three-child policy, which has become a current hot topic in society (51). In addition, the fertility rate of the floating population was lower than that of residents, so it was necessary to pay attention to the fertility situation of the floating population. The birth of the floating population was related to the urban construction and development. However, at present, there are few research studies on the factors affecting the fertility of the floating population, and the corresponding prediction models are also relatively lacking.

This study showed that personal status, the duration of settlement, scope of migration, economic conditions, and social services all influence the reproductive behavior of the floating population. For details, Han new industry workers were more likely to give birth to one child and less likely to give birth to two children than non-Han new industry workers. Migrant farmers were more active in childbearing and have children earlier on average. People with junior high school education were the most likely to have a child, showing a U-shaped pattern that first increased and then decreased. However, in terms of having a second child, the less educated new industry workers were more motivated to give birth. Higher educational attainment was associated with a later age at first birth and a larger spacing between births. Employers were much more likely to have children than blue-collar workers.

New industry workers who had settled for more than 10 years after emigration were more active in their reproductive behavior. The improvement of family economic conditions had a positive influence on the fertility behavior of new industry workers. The influence of monthly income on the second child was less than that of the first child. The new industry workers in first-tier cities were less likely to give birth to a kid and more likely to delay childbearing. New industry workers who owned property locally were far less likely to have a second child. Improvements in insurance and health services might be associated with later age at first birth and longer intervals between births.

A study of women's health in Texas found that an increase in clinics around the house would lead to an increase in fertility (52). At the first International Symposium on West African Studies, experts pointed out that improving the current situation of maternal and child health service supply in China could improve the fertility desire of the population of childbearing age (53). Combined with these studies, it could be concluded that the fertility desire of residents could be improved by bettering social medical services.

The health insurance reform has reduced the cost of pregnancy, which might increase the fertility rate of married women aged 20–34 years by about 1% (54). Insurance services in this study did not have a statistically significant effect on the fertility rate of new industry workers. This might be related to the unsatisfactory social security coverage of Chinese new industry workers (55) and the geographical limitations of some medical insurance (56, 57). Household income correlated closely with the number of children in metropolitan areas of the United States (58). People with better personal economic conditions expected more children. Also, in countries and regions with high economic status, the fertility rate of local women was relatively higher (59). Therefore, it supported the result that the increase in family income could promote reproductive behavior. People with higher education would delay marriage to some extent, resulting in a lower fertility rate (60). The human capital theory suggested that investment in education might produce marriage market returns (61). However, the higher demand for marriage partners among highly educated people, coupled with the huge cost of marriage caused by soaring property prices in China, might have reduced the desire of this group to get married, thus lowering the fertility rate. Consistent with this conclusion, people with higher education backgrounds were less likely to get married than those with a high school diploma, according to the Chinese Family Group Study (62).

A study on the ex-pat effect of a Maya Population from rural Guatemala found that new industry workers had their first babies earlier but had lower fertility rates, which could be attributed in part to stress (63), which explained the negative correlation between the migration range and the age of the first childbearing in this study, to some extent. After settling down for more than 10 years after migration, the immigrants' reproductive behavior was more active. This might be related to their wealth accumulation and improved quality of life.

First-tier cities and high housing prices might be important factors in decreasing fertility rates and delaying childbirth (64, 65). New industry workers who had their own houses in first-tier cities had spent longer time accumulating wealth in the past, thus delaying their childbearing. A study on Korean couples found that families living in non-metropolitan areas and renting houses had more active fertility behavior, which might be related to the family's housing requirements and the length of time spent to meet these demands (65). It was also confirmed by the results of our study. More preferential policies for renting or buying property might provide economic stability for new industry workers' initial settlement and meet their housing needs to promote the fertility rate. The difference between the rural floating population and non-agricultural fertility behavior might be related to the one-child policy of China announced in 1979. The policy was first strictly carried out in Shanghai and other big cities, while the implementation strategy was relaxed in the rural population with certain flexibility (66). Moreover, the concept of “raising children for old age” was deeply rooted in the rural population, and its fertility desire was stronger than that in the urban population.

In terms of employment, employment opportunities in first-tier cities were more attractive to the floating population (67), and it was more necessary to protect the basic rights and interests of new industry workers, such as income, and maintain their employment stability by building harmonious labor relations (68). In addition, it was necessary to improve the affordability of urban housing (69) and bring more new industry workers into the security scope of public rental housing and the community service system. Moreover, welfare policies such as housing subsidies could promote the settlement of the floating population (70). It was also suggested that their enthusiasm be increased to participate in insurance by expanding the coverage of work-related injury insurance (71), endowment insurance (72), and medical insurance (73, 74). Referring to medical and health services, integrating the floating population into the community health services, strengthening the maternal healthcare system, and adjusting the number of subsidies could improve the fertility rate of the floating population (75).

According to the associated factors obtained by the regression models, neural network, naive Bayes, and Logistic regression models were applied to predict the fertility behavior of the floating population. It was found that artificial neural networks and logistic regression could predict marriage and childbearing behavior of the floating population more effectively. This might be related to the assumption that the naive Bayes model needed to satisfy the independence of each feature vector (76).

Logistic regression used the logic function of a linear combination of numerical features to model the logarithmic probability of each category (77). Neural networks had low requirements for data. An artificial neural network consisted of an input layer, a hidden layer, and an output layer, with each linked to an earlier layer and each layer linked to another layer. In this study, we specified a hidden layer, a hyperbolic tangent, as the activation function and the identity activation function of the output layer and determined the model when the optimal difference of fitting conditions between the training set and test set was obtained (78). Among them, the performance of the ANN was superior to other networks in the field of medical prediction tasks (79). Accurate prediction of population fertility could reveal the trend of urban population growth, facilitate urban population management and construction, and benefit social stability and prosperity. Therefore, based on the information on the floating population's identity, the duration of settlement and migration scope, economic conditions, and social services, it was suggested that an artificial neural network and logistic regression be applied to predict fertility behavior, and the model coefficients be updated in time according to real-time data.

The study also had some limitations. The data set of Floating Population Dynamic Monitoring Survey of China in 2016 needed to be further supplemented by longitudinal follow-up data. In the analysis of related factors, regression analysis was used in this study, focusing on the dependence between variables. The causal relationship should be further explored to guide practical application.

In conclusion, the factors related to the reproductive behavior of the floating population were complex, such as social health services, family income, and urban living burden. We recommend the expansion of social health and insurance services, the promotion of employment and income levels of new industry workers, and the introduction of preferential policies for settling down. Furthermore, we should not blindly stimulate marriage and childbearing for the sake of urban population development. Due to the promotion of eugenics and the improvement of social construction such as insurance, people would no longer emphasize the number and speed of birth. Instead, they might pay more attention to the education and cultivation of the next generation. By incorporating the multi-factor analysis, the statistically significant correlation factors of personal status, the duration of settlement after migration and migration scope, economic conditions, and social services could be obtained. The artificial neural network model and logistic model with better performance might be used to make individual predictions. The prediction model of the population's childbearing behavior with high accuracy could help relevant departments to better predict and intervene in the development of the floating population, screen the population with low fertility possibility, and improve their fertility rate, ultimately to alleviate population aging and promote economic development.

## Data availability statement

The data analyzed in this study is subject to the following licenses/restrictions: Application to data provider is required. Requests to access these datasets should be directed to Floating Population Service Center of China National Health Commission, China's Floating Population Dynamic Monitoring Survey Data Set (2016), http://hdl.handle.net/20.500.12291/10227.

## Author contributions

XL and XZ designed the research study. XZ performed the research and wrote the manuscript. XL, XZ, ZZ, LG, LC, YZ, CH, JX, and JL offered help and advice on data collection and analysis. All authors have contributed to editorial changes in the manuscript, read, and approved the final manuscript version.

## Conflict of interest

The authors declare that the research was conducted in the absence of any commercial or financial relationships that could be construed as a potential conflict of interest.

## Publisher's note

All claims expressed in this article are solely those of the authors and do not necessarily represent those of their affiliated organizations, or those of the publisher, the editors and the reviewers. Any product that may be evaluated in this article, or claim that may be made by its manufacturer, is not guaranteed or endorsed by the publisher.
